# Cross-reactive dengue virus-derived monoclonal antibodies to Zika virus envelope protein: Panacea or Pandora’s box?

**DOI:** 10.1186/s12879-018-3572-0

**Published:** 2018-12-10

**Authors:** Shannon A. Gunawardana, Robert H. Shaw

**Affiliations:** 1 Medical Sciences Division, Oxford University, Oxford, UK; 2 Oxford University Hospitals, John Radcliffe Hospital, Oxford, OX3 9DU UK

**Keywords:** Dengue virus, Zika virus, Cross-reactive antibodies, Vaccines, Antibody-dependent enhancement, Cross-neutralisation, Envelope protein, Flavivirus

## Abstract

**Background:**

Dengue Virus (DENV) and Zika Virus (ZIKV) are closely related flaviviruses, circulating in overlapping geographical regions. The recent ZIKV epidemic has been linked to an explosion in reports of microcephaly and neurological defects. It is conceivable that our knowledge of DENV might potentiate the development of a ZIKV vaccine due to the close phylogenetic relationship between these flaviviruses and cross-reactive antibodies, principally to the envelope protein (E protein). Alternatively, cross-reactive antibodies that are generated following vaccination or infection, might become damaging during subsequent infections.

**Main body:**

The aims of this review are to collate and analyse data from a recent series of DENV-derived monoclonal antibody (mAb) panels from different research groups. These panels measured DENV-mAb activity against ZIKV in terms of antibody-dependent enhancement (ADE) and neutralisation. Methodology used across groups was compared and critiqued. Furthermore, the specific antibody targets on E protein were considered and their therapeutic potential evaluated. Shortcomings of hmAb panels suggest ADE may be over-estimated and neutralisation underestimated, as compared to clinical situations. It remains unknown whether preference of enhancement or neutralisation by antibodies to ZIKV E protein is dictated by quantitative aspects of antibody titre or epitope specific variation. Additionally, little is known about how duration between flavivirus reinfections affect secondary antibody response.

**Conclusion:**

This review concludes that our current knowledge of cross-reactive antibodies to E protein is inadequate to anticipate the outcome of deploying an E protein based vaccine to regions co-infected by DENV and ZIKV.

## Background

DENV is the leading arthropod-borne viral infection in the world; [1] estimates predict there are 96 million annual apparent dengue manifestations. [2] Although more than 100 countries are dengue endemic, 75% of DENV cases are localised to Asia-Pacific. [3] In contrast, since 2007 ZIKV epidemics have emerged predominantly in Latin America and the Pacific Islands, accompanied by a troubling increase in Guillain-Barré syndrome in adults and microcephaly in babies born from mothers infected with the virus. [4] The symptomatic presentation of DENV is a spectrum from asymptomatic to dengue shock syndrome. [5] Whereas when symptomatic, ZIKV presentation includes headache, maculopapular rash and febrile illness. [6] Similarities in the presentation of flavivirus infection exacerbate potential misdiagnosis caused by cross-reactive non-specific antibody diagnostic tests.

The *Aedes* genus of mosquito, specifically *Aedes aegypti* and *Aedes albopictus*, is the most common vector of both flaviviruses. [7, 8] Four serotypes exist of DENV (DENV1–4), whereas two strains of ZIKV exist – African and French Polynesian. A key distinction between viruses is that there is apparent inter-strain protection with ZIKV, whereas an individual could be infected by all 4 DENV serotypes in a lifetime. [9, 10] A common flavivirus vector could account for the overlapping DENV and ZIKV endemic regions; this highlights the necessity for greater understanding of cross-reactive antibodies directed to conserved flavivirus epitopes.

E protein is a flavivirus structural glycoprotein that mediates receptor binding and virus-host cell membrane fusion, which is pivotal for enveloped viruses. E protein is composed of three domains with distinct functions – EDI, EDII and EDIII; [10] E proteins display icosahedral arrangement such that 90 E dimers coat the viral surface and switch conformation in relation to virus maturation. [1] The similarities of DENV and ZIKV E protein are highlighted by ~ 55% similarity in amino acid sequences. [11] An interesting distinction of these flavivirus E proteins is the single glycosylation site of ZIKV E protein (Asn 154), whereas DENV E protein has two glycosylation sites (Asn67 and Asn153). [12] Nevertheless, the overwhelming similarity between DENV and ZIKV E proteins permit this glycoprotein to be the major surface protein targeted by cross-reactive antibody binding, as assessed by enzyme-linked immunosorbent assay. [11, 13–15]

## Discussion

### Cross-reactive enhancing antibodies

Antibodies from memory B cells created against DENV or ZIKV during previous infections, may cross-react with other flaviviruses to enhance infection, both clinically and immunologically. This is comparable to antibody-dependent enhancement (ADE) theory, whereby primary DENV infection exacerbates disease severity caused by subsequent heterologous DENV serotype reinfection. [16] Notably, this can occur with antibodies targeted to the conserved flavivirus E protein.

In the last year, several studies have isolated human monoclonal antibody (hmAb) panels from ZIKV and DENV infected donors, to investigate the ability of cross-reactive antibodies to enhance heterologous flavivirus infection. [11, 13–15] Table 1 presents data, that unless otherwise specified, express the ability of hmAbs from DENV-infected donors to enhance ZIKV infection of human cell lines. These data show that cross-reactive antibodies to linear epitopes of E protein, specifically the fusion loop epitope (FLE), are highly enhancing of ZIKV; this epitope is perfectly conserved in the E proteins of ZIKV and DENV2. [11] DENV hmAbs to EDI/II domains were found to be highly cross-reactive against ZIKV, in contrast to the specificity of hmAbs directed to EDIII. [14] A limitation of the most potently enhancing antibody results, 1.6D and D11C, is that measurement of ZIKV infection enhancement used amplification of cell RNA instead of measuring the yield of infectious virus. This is restricted by the assumption that increased RNA correlates to greater virus release from the cell.

**Table 1:** A summary of hmAb experiments investigating ADE of heterologous flavivirus infection

Antibody Name	Concentration (μg/ml)	Target Epitope	Cell line	ADE Measurement	Enhancement Strength
31.3F01 [11]	0.4	Unknown	U937	Flow cytometry	48x
1.6D [13]	20	FLE	K562	qRT-PCR	140x
D11C [13]	20	FLE	K562	qRT-PCR	275x
ZKA3 [14]*	1	EDI/II	K562	Flow cytometry	~75x
ZKA78 [14]*	1	EDI/II	K562	Flow cytometry	~60x
DV82 [14]	1	EDI/II	K562	Flow cytometry	~16x
753(3) C10 [15]	0.1nM**	EDE1	U937	Focus forming assay	~80x
747(4) A11 [15]	1nM**	EDE2	U937	Focus forming assay	~90x
750-2C5 [15]	1nM**	FLE	U937	Focus forming assay	~60x

*qRT-PCR* quantitative reverse transcription polymerase chain reaction

* = hmAbs taken from ZIKV-infected donors and tested for enhancement of DENV infection

** = Molar mass not quoted in paper so unable to convert to μg/ml

~ = Approximation as values read from graph

Limitations of in vitro work are the absence of the complete humoral response; antibodies in vivo might interact with immune system components, such as complement, to augment or suppress enhancing antibodies. Crucially, investigation of the in vivo enhancing ability of the anti-DENV hmAb, DV82, in the 129Sv/Ev immunocompetent mouse model did not increase lethality or symptom severity of ZIKV infection. This implies that cross-reactive hmAbs against EDI/II, namely DV82, enhance ZIKV infection in vitro but not in vivo. Virus tropism might be causal to the discrepancy. However, a more likely explanation is mouse model shortcomings. [14] Immunocompetent mice are less permissive to flavivirus replication; the absence of enhanced infection in vivo could be due to incompatibility of the mouse model and virus, as shown in other work. [17]

An important consideration of the human clinical enhancing antibody response caused by pre-existing, cross-reactive antibodies to ZIKV and DENV is that this response is polyclonal, unlike the monoclonal antibody responses typical of in vitro studies. Investigations of the worst possible scenario of ADE utilise convalescent DENV serum incubation of cells, prior to ZIKV challenge. This had led to inconclusive results, with some studies suggesting that DENV plasma has a potent ADE effect on ZIKV, [13, 15] and others showing that plasma instead has a neutralising effect. [11, 18] A cause of discrepancy could be the duration between infection and extraction of serum, which ranges from 2 months to several years post initial DENV infection. This would affect the quantity of circulating memory B cells against DENV and correspondingly the potency of cross-reactivity. Paul et al. demonstrated that low to medium titres of anti-DENV serum are most potently enhancing of ZIKV. [13] Thus, highlighting that antibody concentration could be causal to contradictory serum studies.

Paradoxically, some hmAbs have been shown to inhibit DENV plasma-mediated ADE of ZIKV infection in vitro, specifically antibodies to the envelope dimer-epitope (EDE) – a quaternary structure that bridges two E protein subunits. In contrast, hmAbs to the FLE have no such inhibitory effect on ADE. [15] Other studies have shown that ‘LALA’ mutants of hmAbs to the EDI/II domain, namely DK82, can also inhibit ADE caused by DENV plasma. [14] This inhibition could be due to hmAbs outcompeting serum enhancing antibodies or perhaps having a neutralising effect. Evidently, the complexities of heterologous enhancement posed by hmAbs and convalescent plasma remain incompletely understood.

### Cross-reactive Neutralising antibodies

In contrast, the panacea of cross-reactivity is the possibility that neutralising antibodies to flavivirus E protein could be exploited to create a ZIKV vaccine. Table 2 presents data from hmAb panel studies regarding the in vitro neutralising abilities of DENV antibodies against ZIKV, unless otherwise specified [18, 19].

**Table 2:** A summary of hmAb experiments investigating neutralisation of heterologous flavivirus infection

Antibody Name	Target Epitope	Cell line	Neutralisation Measurement	EC_50/_PRNT_50_ Concentration (μg/ml)
1.6D [13]	FLE	LLC-MK2	Focus forming assay	Up to 40
D11C [13]	FLE	LLC-MK2	Focus forming assay	Up to 40
ZKA3 [14]*	EDI/II	Vero	Flow cytometry	0.35
ZKA78 [14]*	EDI/II	Vero	Flow cytometry	0.27
2A10G6 [19]	FLE	BHK-21	PRNT	250
752-2C8 [18]	EDE1	Vero	Focus forming assay	8.9 × 10^− 4^
753 (3) C10 [18]	EDE1	Vero	Focus forming assay	3.4 × 10^− 4^
B7 [18]	EDE2	Vero	Focus forming assay	Unknown

*PRNT* Plaque Reduction Neutralisation Test

* = hmAbs taken from ZIKV-infected donors and tested for neutralisation of DENV infection

EC_50_/PRNT_50_ concentrations quoted to 2 significant figures

Intriguingly, the neutralisation potency of antibodies to the FLE vary depending on the cell type in which virions are produced. Insect cells contain high levels of prM, the presence of which better exposes the FLE and leads to greater neutralisation by dengue virions produced in these cells compared to virions produced in human cells. [20] Although 2A10G6 is a DENV serotype cross-reactive antibody, it is unable to fully neutralise virus produced in primary human cells, likely due to low prM expression. Furthermore, such antibodies to the FLE have low cross-reactive neutralisation abilities against ZIKV. [19]

A further target of hmAbs is the novel quaternary epitope - EDE; antibodies to this region have a higher neutralising capacity and lower ADE effect than antibodies to the FLE (Tables 1 and 2). Antibodies targeting the EDE are subdivided based on their requirement of glycosylation for binding; EDE2 antibodies require glycosylation, whereas EDE1 antibodies do not. [21] Focus forming neutralisation assays show EDE2 hmAbs are of lower avidity for ZIKV than EDE1 hmAbs. [18] This corroborates X-ray crystallography data that indicate conservation between alignment of EDE1 contact residues in DENV and ZIKV. [10] A protocol limitation is that Vero cells only detect cell type-specific neutralising responses, as this cell line is poorly permissive to partially mature virions; subsequently, this produces an underestimate of neutralisation. Raji cells expressing DC-SIGNR are preferable for neutralisation investigations as this cell line permits the detection of more representative, cross-reactive patterns of neutralisation. [22]

A shortcoming of hmAb panels, in both neutralisation and enhancement studies, is that these may not fully represent the host antibody repertoire to infection as some antibodies may not survive the immortalisation process. Panels are also limited by uncertainty in accurate diagnosis of previous subclinical DENV and ZIKV infections. Furthermore, hmAb panels only characterise B cells from the blood, whereas splenic B cells are ignored. [23] Antibody sequencing could be employed to detect somatic mutations that identify neutralising antibodies generated during prior infection. [24] Nevertheless, in vitro studies suggest hmAbs against DENV E protein can neutralise ZIKV; however, caution must be taken as this is yet to be demonstrated in vivo in humans.

A vital discriminant between antibodies against EDE and FLE is the maturity of virus to which they bind. Crystallisation screen and X-ray diffraction datasets with molecular replacement suggests that 2A10G6 binds the ZIKV EDII tip at a perpendicular angle, binding immature or partially immature virus. Consequently, antibody binding to FLE is dependent on virion ‘breathing’ that transiently exposes the FLE hidden epitope. [19] In contrast, cryo-electron microscopy studies of an EDE1 antibody, 753(3) C10, (C10) complexed with DENV recombinant E protein dimer show at pH 6.5 (early endosomal pH), C10 locks virus surface E proteins; whereas, at pH 5 (late endosomal pH), C10 locks the E protein raft structure. This suggests C10 prevents structural rearrangement of E proteins during virus-endosome membrane fusion. [18] The ability of EDE antibodies to trigger more potent neutralisation and the EDE1 subclass to bind mature virus forms, suggests that this epitope is the superior vaccine candidate.

### Therapeutic potential for ZIKV

The ability of DENV-derived mAbs that target EDE1, to protect against both DENV and ZIKV is currently being investigated. Studies using the AG129 mouse model show that all C10-protected mice survive following ZIKV challenge, in contrast to 60% mortality in control mice. The C10 mAb is a preferable immunoprophylaxis candidate than FLE mAbs, as only two doses of 10 μg are needed for a protective effect, in contrast to the 500 μg dose of 2A10G6. [25] The potential for C10 mAbs to cause ADE could be reduced by creating a ‘LALA’ mutation, in which the Fc region is mutated to abolish interaction with myeloid cell receptors and prevent enhanced viral uptake; this would potentiate its use in passive immunoprophylaxis of pregnant women at risk of ZIKV. [1]

Promising vaccine candidate models include virus like particles (VLPs) which lack viral genomes and thus are replication defective. Recent work developing subunit vaccines with ZIKV EDIII-displaying VLPs elicited potent humoral responses in immunocompetent C57BL/6 mice. [26]

### Future directions

Several questions remain unanswered regarding cross-reactive antibody neutralisation and enhancement to flavivirus E proteins. To anticipate the effects of ZIKV infection and vaccination in a DENV-infected region, we must first expand our knowledge of cross-reactive antibody responses; thus, I propose the following experiments (Table 3). It is paramount that we optimise in vitro techniques of measuring antibody-mediated neutralisation and enhancement, perhaps through next-generation methods such as green fluorescent protein (GFP) expression in reporter virus particles (RVPs).

**Table 3:** My suggested future experiments. *Own work*

Aims	In Vitro	In Vivo
Utilise a known DENV mAb to create a vaccine that can neutralise both DENV and ZIKV.	Use RVPs to conduct studies in Raji DC-SIGNR and U937 cells to investigate whether DENV and ZIKV stoichiometry determines the quantitative relationship between neutralisation and ADE.	Create a VLP using the EDE1 region to which C10 mAbs are directed to test protection against ZIKV and DENV challenge in a suitable immunocompetent mouse model.
Examine the nature of cross-reactive serum using physiologically relevant antibody titres at varying incubation periods.	Explore the multiple hit hypothesis with immune sera to observe the effects of antibodies binding to multiple antigens. Use molecular modelling and reporter GFP expression in RVPs to measure neutralisation.	Investigate whether ADE by convalescent serum aids the trans-placental transfer of ZIKV in mouse models.

## Conclusion

The pinnacle of cross-reactive neutralising antibody research would be the discovery of a universal immunogen of DENV and ZIKV; presently, EDE1 is a prominent candidate. Shortcomings of hmAb panels suggest ADE may be over-estimated and neutralisation underestimated, as compared to clinical situations. It remains unknown whether preference of enhancement or neutralisation by antibodies to ZIKV E protein, is dictated by quantitative aspects of antibody titre or epitope specific variation. Additionally, little is known about how duration between flavivirus reinfections affect secondary antibody response. The likelihood a ZIKV vaccine will be deployed to areas of DENV incidence indicate that understanding of cross-reactive antibody interplay is vital to an appropriate public heath response. Sanofi’s Dengue vaccine recently sparked safety concerns as data showed DENV-immune participants were at increased risk of severe dengue infection following vaccination. [27] Currently, immunological data is insufficient to prove an ADE mechanism. Thus, it is imperative to further investigate the paradoxical intricacies of cross-reactivite DENV-derived mAbs to create a safe and effective flavivirus vaccine.

## Abbreviations

ADE: Antibody-dependent enhancement
C10: 753(3) C10
DENV: Dengue Virus
E protein: Envelope protein
EDE: Envelope dimer-epitope
FLE: Fusion loop epitope
GFP: Green fluorescent protein
hmAb: human monoclonal antibody
mAb: monoclonal antibody
RVPs: Reporter virus particles
ZIKV: Zika Virus
